# Supplementary material for: Endophytic Bacterial Community, Core Taxa, and Functional Variations Within the Fruiting Bodies of Laccaria
Source: Microorganisms. 2024 Nov 12;12(11):2296. doi: 10.3390/microorganisms12112296 (PMC11596330; doi:10.3390/microorganisms12112296)
Supplement: Supplementary file 1 [file microorganisms-12-02296-s001.zip › Supplementary Figure S1.pdf]

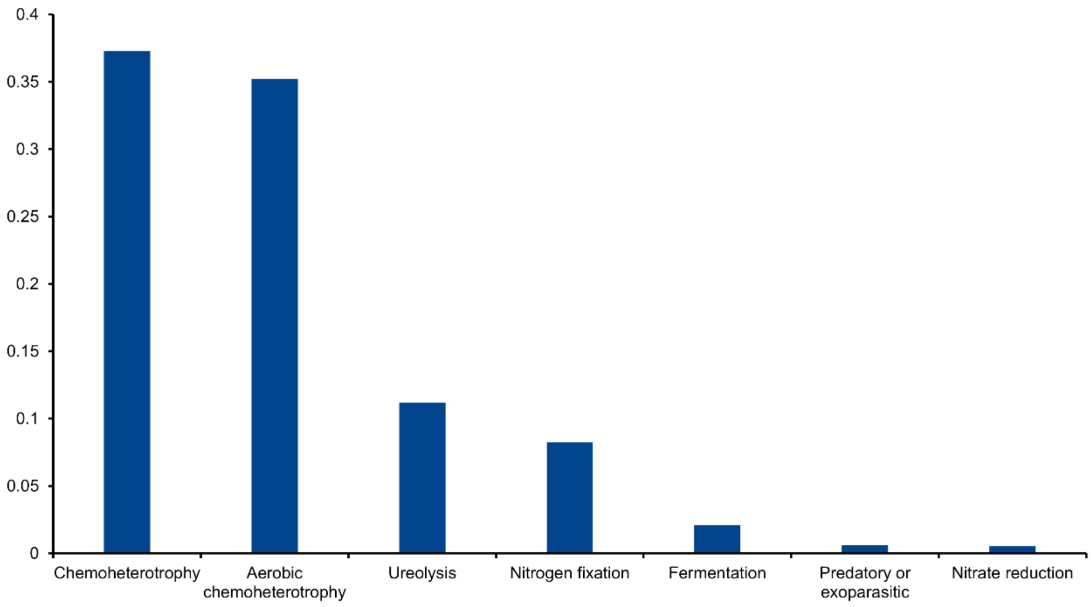

Supplementary Figure S1. *Laccaria* endophytic bacterial community ecological functional abundance prediction (Top 7 in relative abundance) based on Functional Annotation of Prokaryotic Taxa (FAPROTAX) genome database.
