# Supplementary material for: Endophytic Bacterial Community, Core Taxa, and Functional Variations Within the Fruiting Bodies of Laccaria
Source: Microorganisms. 2024 Nov 12;12(11):2296. doi: 10.3390/microorganisms12112296 (PMC11596330; doi:10.3390/microorganisms12112296)
Supplement: Supplementary file 1 [file microorganisms-12-02296-s001.zip › Supplementary method.pdf]

The method for the DNA extraction kit

Procedures:

1. Take about 100 mg of fresh tissue and add liquid nitrogen to grind it thoroughly.
2. Transfer the milled powder quickly into a centrifuge tube pre-filled with 700μl of 65°C pre-warmed Buffer GP1, quickly invert it to mix well and place the tube in a 65°C water bath for 20 min, inverting the tube to mix the samples several times during the water bath.
3. Add 700 μl chloroform, mix thoroughly and centrifuge at 12, 000 rpm for 5 min.
4. Carefully transfer the upper aqueous phase from the previous step into a new centrifuge tube, add 700 μl of Buffer GP2 and mix well.
5. Transfer the homogenized liquid to spin columns CB3 and centrifuge at 12, 000 rpm for 30 sec, discard the waste liquid.
6. Add 500 μl of Buffer GD to the spin columns CB3, centrifuge at 12, 000 rpm for 30 sec, pour off the waste solution, and put the spin column CB3 into a collection tube (2 ml).
7. Add 600 μl of Buffer PW to the spin column CB3, centrifuge at 12, 000 rpm for 30 sec, pour off the waste solution, and place the spin column CB3 into the collection tube.
8. Repeat step 7.
9. Place the spin column CB3 back into the collection tube, centrifuge at 12, 000 rpm for 2 min, pour off the waste liquid. Leave the spin column CB3 at room temperature for a few minutes to thoroughly dry the rinse solution remaining in the adsorbent material.
10. Transfer the spin column CB3 into a clean centrifuge tube, add 50-200 μl of elution Buffer TE dropwise to the middle of the adsorbent membrane overhang, leave at room temperature for 2-5 min, centrifuge at 12, 000 rpm for 2 min, and collect the solution into a centrifuge tube.
